# Instinct of Animals

**Published:** 1887-10

**Authors:** 


### INSTINCT OF ANIMALS.

Some years ago the humorous editor of the *New York Daily Times*, expressed his dissatisfaction with the honey bee, because for thousands of years it had made no improvement in its methods of gathering and storing honey. The honey-comb of centuries ago, was the exact prototype of the honey comb of to-day. In all these centurics of time there had been no alteration, no change for the better in the architectural designs of its store houses. It would indeed be a fruitless endcavor of the most skillful architect to improve upon the design of the honey cell, as produced by the industry of this diminutive llttle worker, whose only guide is the innate faculty with which it is endowed, a faculty which is the same yesterday, to-day and forever. Every succeeding generation of animals of the same species, will have the identical characteristics of their progenitors, be they what they may. The bee, the ant, the beaver—indeed all the various creations which go to make up the animal kingdom, have their distinctive characteristics common to all of a class, which no amount of training in an opposite direction is able to diminish much less to eradicate

Take for example the several varieties of swallows. They all have some instincts in common, such as being migratory, and feeding upon insects which they take upon the wing; but some build their nests in barns and old out-buildings high up under the roof, others under the exterior eaves, others in the chimneys of dwellings, and others still, by boring into the perpendicular sides of sand banks, each family unerringly after its own fashion. Then there is the woodpecker, with its long bill and spear-like tongue. His instinct leads him to feed upon the borers that infest the trunks of trees: with his bill he drills a sure passage way, and spearing his victim upon the point of his tongue, drags him to the feast.

The dog has become familiar to us from an intimate acquaintance of very many years. It is the only animal that forms an attachment for man superior to his own kind, and very many are the tales of his devotion to his biped master, some of which are very pathetic and full of interest. Most of these animals follow in the chase by a keen and unerring scent, whilst some few go altogether by sight. This comes of a natural instinct, but beyond this, very much depends upon education. What then is that faculty in animals, which is susceptible of being cultivated and taught to obey the commands of a master, the pointing of his finger or the waving of a friendly hand? It certainly is not instinct merely, for that rises only to the level of traits, common to particular generi, or to a special order or genus, for example the hound, spaniel, mastiff, setter, shepherd, collie, terrier etc., all which by association with man, take on traits, which, if not superior to instinct, are no less remarkable, and what is of more importance, the same traits are transmitted to their off-spring. It is by this means that some valuable characteristics have been perpetuated.

Such of our readers as have attended a trained animal show, be it of dogs, horses, monkeys, birds, or all of them together, will appreciate what we say. It certainly is not a natural trait for sparrows to play soldier, fire off cannon and make believe dead; nor for dogs to climb ladders, waltz on two legs, and balance themselves on their fore feet; nor for horses to indulge in jokes on a level with a ring master; nor for pigs to play at cards with cunning and skill; but all these things are done in a most satisfactory way to the great amusement of our juvenile classes.

It was only the other day, we read accounts of two horses which led us to the foregoing reflections. One was of a little French pony, at Higgins' Lake, Michigan, which swam to the assistance of a woman and child in imminent peril of drowning, and brought them safely to land, whilst they clung to his mane.

The other is that of a little mare, named Whitefoot, owned by a farmer of New York State. Every school-day morning she takes the two little girls of her master to school, returning with the carriage by herself. At the close of school hours, she makes her appearance with the empty carriage, and takes her little charges home again, being always careful as well as expert in passing vehicles on the road. On a recent Sunday evening, a servant of her master drove Whitefoot to a neighboring village nine miles distant, where he was to take the cars. Tacking a notice to the wagon seat, asking that the mare be suffered to proceed homeward, he turned her head that way, and gave her the rein, and she made the whole distance in about one hour, in good order. No wonder the late Henry Ward Beecher said in the course of one of his sermons, that he had seen many a horse more deserving of heaven than its master.

